# A Preliminary Study of Biliary Microbiota in Patients with Bile Duct Stones or Distal Cholangiocarcinoma

**DOI:** 10.1155/2019/1092563

**Published:** 2019-09-25

**Authors:** Bingrong Chen, Seng Wang Fu, Lungen Lu, Hang Zhao

**Affiliations:** Department of Gastroenterology, Shanghai General Hospital, Shanghai Jiao Tong University School of Medicine, 200080, China

## Abstract

**Background and Objective:**

The distal cholangiocarcinoma (dCCA) is associated with many factors: genes, environment, infection, etc. The current changes in biliary flora are thought to be involved in the formation of many gastrointestinal tract (GIT) diseases, like colon adenocarcinoma. Therefore we want to investigate whether the dCCA has a certain correlation with biliary microecology, and to detect specific strains.

**Methods:**

A total of 68 adults were enrolled, of whom 8 with dCCA, 16 with recurrent choledocholithiasis, and 44 with the onset of common bile duct stones. Endoscopic Retrograde Cholangiopancretography (ERCP) was utilized to collect bile samples for DNA extraction and 16S rRNA gene sequencing, followed by analysis of bile microbiota composition.

**Results:**

First, Proteobacteria, Firmicutes, Bacteroidetes, and Actinobacteria are the most dominant phyla in the bile of patients with dCCA and the onset of common bile duct stoes. Secondly, compared with the onset of common bile duct stones patients, we got a significant increase in the phylum Gemmatimonadetes, Nitrospirae, Chloroflexi, Latescibacteria, and Planctomycetes in dCCA patients. Finally, at the genus level, we obtained sequencing results of 252 bacterial genera from patients with dCCA, recurrent choledocholithiasis, and the new onset of common bile duct stones, revealing heterogeneity among individuals.

**Conclusion:**

To the best of our knowledge, this is the first study of the dysbiosis of bile flora in patients with dCCA. This micro-ecological disorder may be a decisive factor in the formation of dCCA. At the same time, for the first time, this study provides a test chart of biliary microbial populations that may be associated with recurrent choledocholithiasis. The compositional changes of the core microbial group of the biliary tract have potentially important biological and medical significance for the microbiological biliary disorders of dCCA.

## 1. Introduction

Cholangiocarcinoma (CCA) includes intrahepatic, perihilar, and distal CCAs according to the anatomic origin of the tumor. Three subtypes have different clinical behaviors and molecular patterns. As the most common biliary tract malignancy, CCA has a dismal prognosis. Only 10–15% of all patients with CCA are amenable to curative surgery at the time of diagnosis. Majority of patients present at an advanced stage owe to the lack of effective screening strategies [1]. During the past 40 years, the incidence rate of CCA increased significantly in American, with the subtype of intrahepatic CCA dramatically increasing from 0.44 to 1.18 cases per 100,000 person-year [2]. In China, there is no publicly available data about the incidence rate so far, and the median overall survival period is reported to be around 20 months, and the 5-year cancer cause-specific survival varies from 0% to 20% among different subgroups [3]. Given this high fatality rate, and the silent progression of early disease, identifying risk factors for the prevention and early detection of biliary tract cancer is critical to reducing its mortality. Genomic factors, chronic inflammation, biliary cysts, virus are all causes of CCA, but none of them can be assumed as explicit motivation of a certain subtype [4]. Exploration into the mechanism of carcinogenic effects of mutation promotes the newly development of anticancer drugs. However, the total outcome is not as promising as expected due to the highly heterogeneity of CCA. Hepatolithiasis is regarded as a risk factor of CCA, and after hepatic resection for treatments of both stones and strictures in bile duct, tumors still arise in some patients within some years after surgery [5]. No causal association of common bile duct (CBD) stones and distal CCA is described so far, but phenomenal relation of choledocholithiasis and proximal malignant obstruction was reported before [6].

Microbiota now is considered as an irreplaceable composition of the human body. They are indispensable for specific physiological function of the human body. At the same time, a large variety of diseases are thought to have a correlation with gut microbiome. Microbiota modulate carcinogenesis through altered composition of its components (dysbiosis), harmful properties of some bacteria, shift in local distribution of communities, and change in bacterial metabolic activity. Dysbiosis and a defect in host defense cause bacterial translocation leading to inflammation and the activation of TLRs in different cells by microorganism-associated molecular patterns (MAMPs) [7]. Bacteria may cause genotoxic effects (ie., colibactin) which will damage host DNA and activate signaling cascades. The resulting chromosomal aberrations and translocation of microbial process will lead to the activation of IL-23-producing myeloid cells which will promote tumor growth [8]. Another analysis of the whole microbiota revealed differences between extrahepatic cholangiocarcinoma (ECCA) and benign biliary pathology (BBP): the relative proportions of Fusobacteria, Acidobacteria, and Planctomycetes were significantly higher in ECCA [9]. Microbiota differences have been observed in other gastrointestinal tumors like colon adenocarcinoma, where an increasement of Fusobacterium, Prevotella, and Campylobacter, and reduction of short chain fatty acids (SCFA) producers may represent a pattern that distinguish colon adenocarcinoma patients [10]. Of note, Fusobacterium, Prevotella, and Campylobacter were three genera that Francisco Avilés-Jiménez also found significantly more frequently in ECCA, further suggesting they might have a role in gastrointestinal cancers, probably by inducing an unregulated inflammatory response [9]. An interesting fact is that microorganism is detected in more and more organs where they are expected to be sterile before. Female reproductive tract and human urinary are all proved to be a place of residence for microorganism under normal conditions of the body [11, 12]. Traditional opinion is challenged so frequently as to no surprise will be anticipated if new proof of residence is found in other clean region in human. Bile duct has already been proved to carry bacteria under disease condition. The relationship of chronic inflammation of the bile duct and gut microbiota has been investigated in PSC patients [13]. Recurrence of bile duct stones is also ascribed to migration of bacteria from the small intestine. However, whether there is microbiome in the bile duct of healthy individuals and patients without infectious disease is not known yet. In the light of the ethnic dilemma to obtain bile from healthy people, we enrolled dCCA patients and choledocholithiasis patients to do a preliminary research of microbiota in the biliary system.

Recent advances in the technology (e.g., a cost-effective method based on culture-independent 454 pyrosequencing of the bacterial 16S rRNA gene) used to identify and to analyze components of the microbiome have substantially improved our understanding of the microbial communities associated with this human disease [14]. In this study, we undertook a large-scale molecular analysis of 16S rRNA sequences in order to gain a clearer picture of three crucial issues: (1) the structure and component of biliary tract microbial communities in patients with dCCA; (2) the characteristics of biliary tract core microbiome and its potential connection in the formation of dCCA; and (3) the disorder of biliary microbiota with recurrence of common bile duct stones.

## 2. Material and Methods

### 2.1. Patients Enrollment

The study conformed to the ethical guidelines of the 1975 Declaration of Helsinki and was approved by the Institutional Review Board of Shanghai General Hospital.

From February 2016 to January 2017, patients who *were in the process of being* performed ERCP with a diagnosis of distal CCA or CBD stones were recruited to the study in Shanghai General Hospital. If they met the inclusion criteria, informed consent will be obtained for enrollment. For patients with a diagnosis of dCCA, the inclusion criteria are as follows, diagnosis of distal CCA with evidence of abdominal imaging including abdominal ultrasound, computed tomography or magnetic resonance imaging. Exclusion criteria for dCCA patients are as follows: sign of CBD stones, caroli disease, PSC, and previous cancer diagnosis. Patients with diagnosis of dCBD stones were further categorized into two groups, one group is patients who were diagnosed as common bile duct stones for the first without a history of ERCP, and the other group is patients who had recurrent common bile duct stones and had been performed sphincterotomy during previous ERCP procedure.

All of the patients were enrolled into the dCCA group(Tumor group, T), new onset of CBD stones group (CBD stones group, C), and recurrent CBD stones group (Post-ERCP CBD stones group, P). Important past history and main laboratory results were collected and compared among three groups.

### 2.2. Collection of Samples

ERCP were *conducted* in a dedicated ERCP room. Ultraviolet disinfection was applied 30 min before each ERCP operation. Side-viewing endoscopes (TJF240/JF-260V; Olympus Optical, Tokyo, Japan) were used which were strictly sterile before operation to keep its working channel sterile. New plugs for the working channel were applied to every patient. During standard ERCP procedure, the bile samples were collected through a sterilied catheter which went through the working channel into the bile duct. After successful intubation of the catheter into the bile duct, the tip of the catheter should be in the lower third of CBD, which was confirmed under fluoroscopy. Then the bile was aspirated out through the catheter into a 10 ml syringe, and immediately put into −80°C refrigerator for storage. 5–10 ml of bile was necessary for each patient.

### 2.3. DNA Extraction and MiSeq Pyrosequencing of 16S Ribosomal RNA Gene Amplicons

#### 2.3.1. DNA Extraction and Quality Control

DNA was purified and stored by standard methods [15]. Total DNA was extracted from each noncentrifuged sample with the OMEGA DNA Kit (Omega Bio-tek, Norcross, Georgia, USA) following manufacturer's instruction. A Qubit picogreen Fluorescence quantification system was used to quantify all the DNA, and 1% agarose gel electrophoresis was used to examine the DNA quality.

#### 2.3.2. 16S rRNA Amplicon Sequencing

Specific primers with barcode were made according to the region to be sequenced. To ensure the accuracy and consistency of data analysis, low cycle number amplification is used to ensure that the cycle number of sample amplification is consistent. TaKaRa EX Taq (Takara Bio, Mountain View, CA, USA) DNA polymerase is used for PCR (Applied Biosystems 2720 Thermal Cycler). The amplified products were identified by 2% agarose gel electrophoresis. The AxyPrep Mag PCR Clean-Up Kit (Thermofisher scientific, CA, USA) magnetic beads were recovered and purified, and then utilized for quantitative construction. Detailed protocols for the two-step 16S rRNA gene amplification and library construction procedures are reported before [16, 17].

### 2.4. Data Analysis

Ages were presented as mean ± SD. Gender differences were assessed using Chi-squared-tests. Comparison of past history and laboratory results among three groups were done by using Chi-squared-test. *Comparison of the relative abundance of microbiota among three groups were done by nonparametric tests. *Alpha diversity is used to analyze the species diversity of individual samples, measured by species richness (Richness, Chao, ACE) and diversity index (Shannon, Simpson). Linear discriminant analysis effect size (LEfSe) was introduced to identify bacterial biomarkers for the three groups. Differences with *P* < 0.05 were considered to be statistically significant.

## 3. Results

### 3.1. Characteristics of Patients

Totally 68 patients were enrolled in the study including 8 with dCCA (5 males, 3 females), 44 with the new onset of common bile duct stones (18 males, 26 females), and 16 with recurrent choledocholithiasis (9 males, 7 females). There was no significant difference among these three groups in regard of average age, proportion of gender, co-existing chronic disease, presence of gallbladder stone, and abnormal ratio of laboratory tests (Table 1).

### 3.2. Increase in Biliary Microbial Diversity Is Closely Related to the Presence of dCCA

We compared the richness of the bacterial community (Chao index) and diversity (Shannon index) in the bile of patients with dCCA and patients with common bile duct stones (Table 2). We observed no difference in the Chao index among the three groups. The Chao index of Group T, Group C, and Group P is 243.42 ± 73.27, 202.26 ± 94.64, and 181.2 ± 71.31 separately. Comparing to the other two groups, the Shannon index for dCCA patients (2.88 ± 1.19) was significantly higher than that of Group C (1.95 ± 1.11) (*P* = 0.36) ([Supplementary-material supplementary-material-1]).

**Figure 1:**
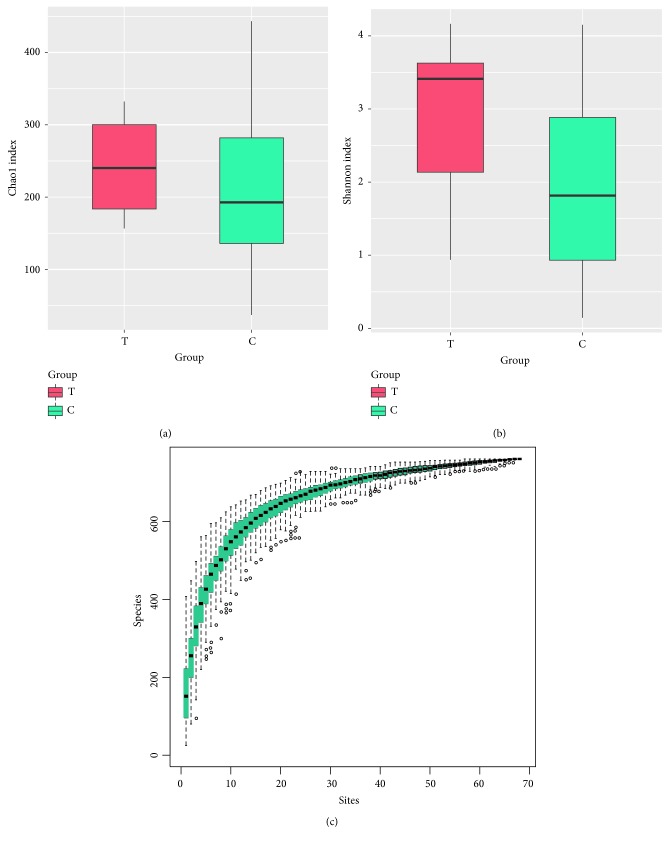
The richness and diversity of biliary microbiota in patients with dCCA and the onset of common bile duct stones(a&b). Richness rerefaction plot from all samples,the abscissa indicates the number of sequences randomly extracted from the sample, and the ordinate indicates the number of OTUs obtained by clustering the corresponding sequence numbers (c). The dCCA group (Tumor group, T) and the new onset of CBD stones group (CBD stones group, C) denoted as “Group T” and “Group C”, respectively, in the figure.

The results of the Specaccum species (Figure 1(c)) accumulation curve show that as the sample size increases, the curve shows a sharp rise, indicating that a large number of species are found in the community, as the number of test samples continues to increase, the curve tends to be flat, indicating that species in this environment do not increase significantly as the sample size increases. This curves shows that the experiment is adequately sampled and data analysis can be carried out.

Similar richness (*P* = 0.24, Figure 1(a)) and higher diversity (*P* < 0.05Figure 1(b)) measures were observed in patients with dCCA and the new onset of common bile duct stones.

### 3.3. Relative Taxon Abundance in the Microbiota of Patients with dCCA and the New Onset of Common Bile Duct Stones

**Figure 2:**
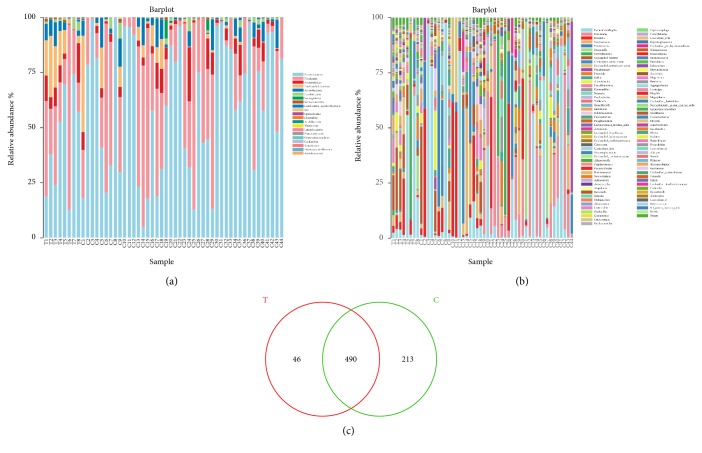
Relative taxa abundance and OTUs comparison in patients with dCCA and the onset of common bile duct stones. The overall microbial composition at the phylum (a) and genus (b) level illustrated that the taxonomic composition varies widely among individuals, but Proteobacteria and Firmicutes were the dominant phylum among all individual, and Escherichia/Shigella and Halomonas were the major genus. It indicated that the core OTUs from patients with dCCA and the new onset of common bile duct stones were composed of 536 and 703 OTUs, respectively, of which 490 were shared between the two groups (c). The dCCA group (Tumor group, T) and the new onset of CBD stones group (CBD stones group, C) denoted as “Group T” and “Group C”, respectively, in the figure.

We explored the biliary microbial community characteristics of patients with dCCA, and compared the relative abundance of microbiota between patients with dCCA and the new onset of common bile duct stones. The overall microbial composition at the phylum and genus level illustrated that the taxonomic composition varies widely among individuals, but Proteobacteria and Firmicutes were the dominant phylum among all individuals (Figure 2(a)), and Escherichia/Shigella and Halomonas were the major genus (Figure 2(b)).

The core bacterial community at the OTU level was shared by at least 50% of the samples in each group and was shown in the Venn diagrams constructed, to evaluate the shared OTUs in patients with dCCA and the new onset of common bile duct stones (Figure 2(c)). It indicated that the core OTUs from patients with dCCA and the new onset of common bile duct stones were composed of 536 and 703 OTUs, respectively, of which 490 were shared between the two groups.

**Figure 3:**
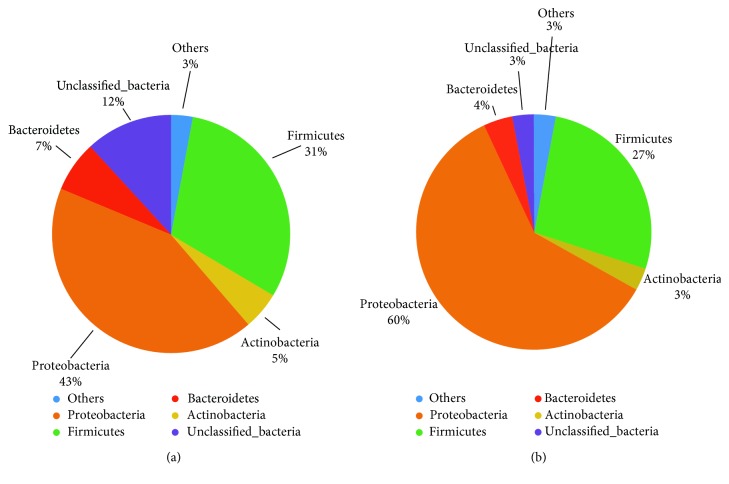
Relative abundances of dominant phyla in patients with dCCA (a), and the onset of common bile duct stones (b), patients from both groups contained five dominant phyla: Proteobacteria, Firmicutes, Bacteroidetes, Actinobacteria, and Unclassified_Bacteria, which accounted for 97% of the biliary microbes. Relative abundance of Proteobacteria, Firmicutes, Bacteroidetes and Actinobacteria is similar between patients with dCCA and the new onset of common bile duct stones. The dCCA group (Tumor group, T) and the new onset of CBD stones group (CBD stones group, C) denoted as “Group T” and “Group C”, respectively, in the figure.

We compared the biliary microbial community of patients with dCCA and the new onset of common bile duct stones. Patients from both groups contained five dominant phyla: Proteobacteria, Firmicutes, Bacteroidetes, Actinobacteria, and Unclassified_Bacteria, which accounted for 97% of the biliary microbes. Relative abundance of Proteobacteria, Firmicutes, Bacteroidetes, and Actinobacteria is similar between patients with dCCA and the new onset of common bile duct stones (Figure 3).

Besides abundance of Gemmatimonadetes, Nitrospirae, Chloroflexi, Latescibacteria, Unclassified_Bacteria, and Planctomycetes (*P* < 0.05) increased in patients with dCCA, and notably Gemmatimonadetes, Latescibacteria, and Nitrospirae were not detected in the samples from patients with the new onset of common bile duct stones (Table 3).

At the genus level, 252 genera were identified; the five genera with the highest relative abundances in patients with dCCA were Escherichia/Shigella, Staphylococcus, Klebsiella, Unclassified_Enterobacteriaceae, and Faecalibacterium, whereas those inpatients with the new onset of common bile duct stones were Escherichia/Shigella, Halomonas, Klebsiella, Streptococcus, and Enterococcus ([Supplementary-material supplementary-material-1]). And the abundance of Staphylococcus, Okibacterium, and Corynebacterium (*P* < 0.001) is more abundant in patients with dCCA (Table 4).

### 3.4. Biliary Microbiota Variability and Its Association with Biliary Disease

**Figure 4:**
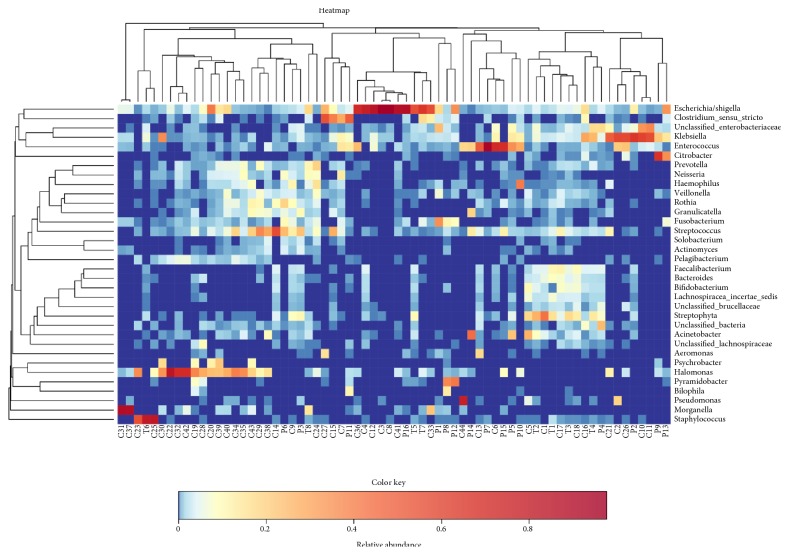
Heatmap of bile microbial from all samples. We tested bile in patients with biliary tract tumors and compared all bile microbial organisms in patients with biliary tract tumors, recurrent choledocholithiasis, and the new onset of common bile duct stones. Analysis of bile microbial colonies reveals heterogeneity between individuals. The dCCA group (Tumor group, T), the new onset of CBD stones group (CBD stones group, C), and recurrent CBD stones group (Post-ERCP CBD stones group, P) denoted as “Group T”, “Group C”, and “Group P”, respectively, in the figure.

We tested bile in patients with biliary tract tumors and compared all bile microbial organisms in patients with dCCA, the new onset of common bile duct stones, and the recurrent choledocholithiasis. A total of 252 bacterial species were identied, clustering was not consistent with patient grouping ([Supplementary-material supplementary-material-1]). Analysis of bile microbial colonies with an abundance  ≥0.2% reveals heterogeneity between individuals (Figure 4). Rough literature mining, Cluster II contained most of the intestinal microbes, whereas Cluster I contained most of the oral cavity and environmental inhabitants. High within-sample abundance was observed for some species, such as Pseudomonas (77.39%) in C44 and Citrobacter (66.52%) in P9. Escherichia/Shigella, Klebsiella, Enterococcus, Haemophilus, and Streptococcus were the species identied in almost all individuals ([Supplementary-material supplementary-material-1]). The current changes in biliary flora are thought to be involved in the formation of common bile duct stones and the recurrence of calculi after ERCP. Therefore, we want to prove that the recurrence of calculi after ERCP has a certain correlation with biliary microecology. We applied LEfSe analysis to further identify the significantly different abundance between patients with recurrent choledocholithiasis and the new onset of common bile duct stones.

**Figure 5:**
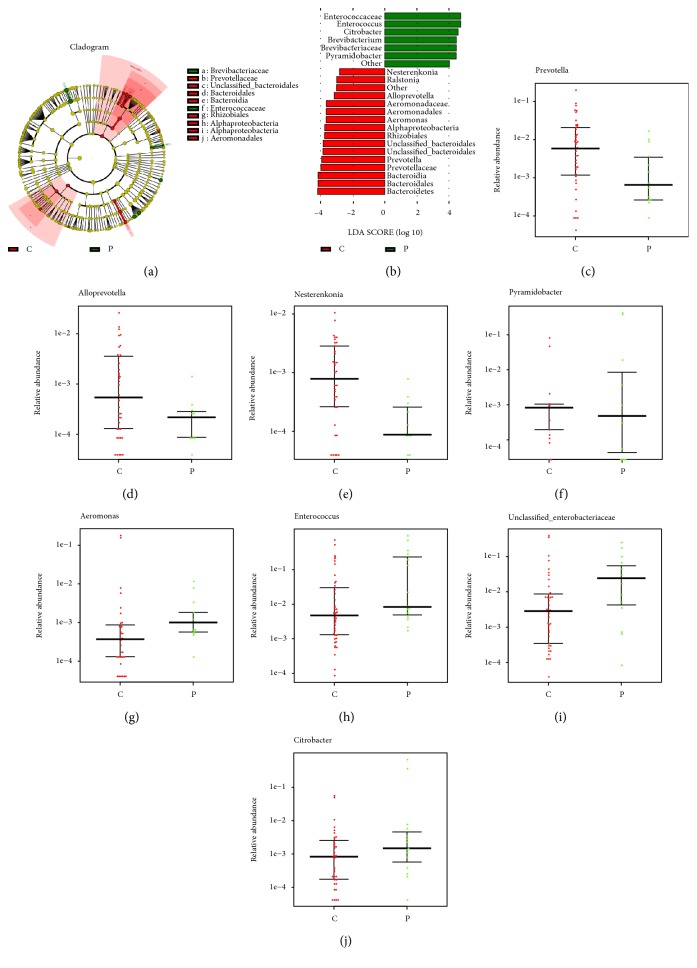
Characteristics of microbial community composition in patients with recurrent choledocholithiasis and the onset of common bile duct stones. (a) Cladogram representing taxa enriched in biliary microbiota community of the two groups detected by the LEfSe tool. Differences were represented by the color of the most abundant class (red, patients with the onset of common bile duct stones; green, patients with recurrent choledocholithiasis). The diameter of each circle is proportional to the taxon's abundance. The central point represents the root of the tree (Bacteria), and each ring represents the next lower taxonomic level. (b) Histogram of the LDA scores computed for different abundance levels between patients with recurrent choledocholithiasis and the onset of common bile duct stones, as detected by the LEfSe tool. (c–j) Relative abundance of Prevotella, Alloprevotella, Nesterenkonia, Pyramidobacter, Aeromonas, Enterococcus, Unclassified_Enterobacteriaceae, and Citrobacter in biliary microbiota community of patients with recurrent choledocholithiasis and the onset of common bile duct stones.

A cladogram (Figure 5(a)) was used to represent the predominant bacteria and the structure of the microbiota in both groups. LEfSe analysis revealed 23 taxa, distinguishing biliary microbial communities of patients with recurrent choledocholithiasis and the new onset of common bile duct stones by a LDA score above 2.8. In these taxa, 7 and 16 were identified as enriched within the patients with recurrent choledocholithiasis and the new onset of common bile duct stones, respectively (Figure 5(b)). At the genus level, abundance of Prevotella, Alloprevotella, Nesterenkonia, and Pyramidobacter (*P* < 0.05) increased in patients with the new onset of common bile duct stones (Figure 5(c)–5(f)), whereas Aeromonas, Enterococcus, Unclassified_Enterobacteriaceae, and Citrobacter (*P* < 0.05) were more abundant in patients with recurrent choledocholithiasis (Figures 5(g)–5(j)).

## 4. Discussion

Existence of microbiota in healthy bile duct is not acceptable widely as a matter of fact till now. Inner part of the body should be free of microorganisms under no infectious condition. Due to the ethical dilemma to collect bile on normal recipients, we selected dCCA patients as research subjects, who are supposed to be without clinical inflammation. Since microbiota can be found in human bile duct, another question we always want to figure out is what's the relation of them with the microform in the gut. The potential relationship of bile duct microbiota and the disease itself is also of our interest. The role of gut bacteremia in the formation of the CBD stones has been discussed before [18–20]. Infection of the gallbladder and migration of germ is also a proven origin of CBD habitants. Oddi's sphincter is an inborn barrier between intestine and bile duct. However, during the process of ERCP for CBD stone patients, sphinterectomy is probably performed to achieve total removal of calculi. Therefore the reflux of bacteria from the gut to the bile duct is inevitable in addition to the imaging evidence of reflux of content of duodenum [21]. High recurrence rate of CBD stones after ERCP was partially ascribed to the anatomic change of protection [22].

By using a targeted amplicon sequencing approach for 16s rRNA, our study demonstrates an underlying biliary microbiota dysbiosis present with dCCA. This is the first study to use 16S rRNAs to clarify the composition of biliary tract microbiota in patients with dCCA and compare them with patients with the new onset of common bile duct stones. Methods such as Alpha diversity analysis can identify differences of microbial components and diversity both between and within the biliary tract microbiota from patients with cancer background and without cancer background.

Our results discovered, in terms of species diversity, biliary tract, like the intestine, has highly abundant microbial colonies. We also discovered that biliary tract microbiota has the heterogeneity between individuals. The reason may be explained by the complex source of bacteria including other parts of gut and mouth. Under pathological condition, the connection of bile germ and intestine was also confirmed. A high microbial diversity in the bile duct was recently reported in the only other study in humans, although that was done in patients with cholesterol gallstones [18]. In addition, whereas in a previous research Proteobacteria comprised around 30% of phyla in the stomach [23], in the bile duct Proteobacteria represented 60%, which is more similar to values described in the small intestine [24]. Surprisingly though, in our research Proteobacteria, Firmicutes, Bacteroidetes, and Actinobacteria, which account for 97% of the biliary flora, are all intestinal flora. This raises a possibility that biliary tract microbiota could be originated from the duodenum. Further evidence is obviously needed to explore this possibility. If is there any possibilities those bacteria are from the small intestine is unknown. For tumors located near to the orifice of the bile duct, it will probably affect the function of the sphincter of Oddi. However, obstruction will definitely lead to the increasing of inner pressure of the proximal duct.

Our research also confirmed significant changes in biliary microbial components between patients with dCCA and patients with first episode of common bile duct stones. Within the biliary tree of patients with dCCA, there exists an over growth of the bacterial phylum Gemmatimonadetes, Nitrospirae, Chloroflexi, Latescibacteria, and Planctomycetes. Planctomycetes are a set of fastidious Gram-negative bacteria related to Verrucomicrobia and Chlamydia in a so-called “PVC (Planctomycetes-Verrucomicrobia-Chlamydiae) superphylum” [25]. These organisms are mainly environmental bacteria found in water sources, but recent searches detected DNA sequences specific for Planctomycetes in the digestive tract of healthy individuals [26]. A study found Planctomycetes DNA in 2 out of 100 blood samples from patients suffering from leukemia with neutropenia induced by chemotherapy, as well as fever, rash, pneumonia, and diarrhea. Therefore they suggest antibiotic-resisting Planctomycetes may be pathogenic in these patients [27]. The phylum Gemmatimonadetes was present in both the sediment and microbialite contigs, and comprised 7-8% of the protein coding open reading frames (ORFs) [28]. The Gemmatimonadetes contigs annotated mainly as hypothetical proteins; however, positive Gemmatimonadetes annotations included ATPases, Zn-dependent peptidases, and glucose/sorbosone dehydrogenase-like genes. Glucose/sorbosone dehydrogenases transform various sugar moieties into vitamins, including L-ascorbic acid (vitamin C), or can make D-glucono1,5-lactone from D-glucose, which can acidify the extracellular environment, which may lead to the dissolution of carbonate by heterotrophic process [29–31]. However, whether the bacteria have the same physiological function in the intestine is worthy further study. Nitrospira, one of microorganisms derived from biofilm in oligotrophic environments, was detected from dental unit water system (DUW) [32]. Chloroflexi was significantly different between patients with adenomas and healthy volunteers, supporting that colorectal pre-neoplastic lesion maybe the most important factor leading to alterations in bacterial community composition [33]. Changes in microbiota have also been reported in mouth and esophagus cancers [34–36].

Previous studies demonstrated an association between biliary microbiota dysbiosis and human diseases, e.g., cholesterol gallstones [18–20], validating the biliary microbiota dysbiosis may be a key contributor to the presence of biliary diseases. In a compromised and obstructed bile duct, bacteria may find a more favorable environment to colonize. Our results found Bacteroidetes accounted for 7% of bile bacterial colonies from patients with dCCA, partially supporting the above inference. Second, Tumors may alter the biliary microenvironment, implying that the dCCA themselves may actually aggravate the over growth of bacteria in the biliary tract.

The biliary microbiota showed significant inter-individual variation in our test results. For example, the highest abundance of Proteobacteria phylum in one dCCA patient was 74.27% and the lowest was 12.83% in another ([Supplementary-material supplementary-material-1]). High person-to-person variation in biliary tract microbiota could be related to a number of factors: host diets, lifestyles, genotypes, disease status, etc. Aside from interpersonal variation, there are several potential factors that may affect the colonization or survival of bacteria in biliary tract of dCCA patients. First, MDR efflux pump proteins expressed by bacteria can produce bile resistance, allowing bacteria to survive in their ecological niche [37–41], as well as BSH activity [42] produced by bacteria that can protect the bacterial cells which produce it from the toxicity of conjugated bile salts.

In conclusion, we reported for the first time the microbiota in the biliary tract in cancer and non-cancer conditions, and found a significant increment in the phylum Gemmatimonadetes, Nitrospirae and Planctomycetes, and a reduction in Chloroflexi in dCCA. We recognized the need for larger studies to confirm our observations. It remains to be determined if these OTUs have a role in carcinogenesis or if they result from changes in the microenvironment of dCCA. We reported the presence of bacteria commonly described in the environment but rarely in humans, such as Latescibacteria isolated from groundwater and temperate freshwater [43]. We then compared biliary microbiology from patients with recurrent choledocholithiasis and the new onset of common bile duct stones. Notably, all the bacteria detected in the bile of patients with the new onset of common bile duct stones were identified in the bile of patients with the recurrent choledocholithiasis, and there is no significant difference between the Shannon index and the Chao index ([Supplementary-material supplementary-material-1]). We suspect that the mechanism by which the flora causes recurrence of stones may be the change of dominant bacteria, not changes in the entire biliary biological population.

To the best of our knowledge, this is the first study to discover a potential association of the biliary microbiota dysbiosis that is present among dCCA patients. Likewise, our characterization of the biliary tract core microbiome provides potentially significant biological implications about both the unexpected diversity of the microbiome among patients with dCCA, as well as the probable roles of bacteria in the formation of dCCA. Our further research on the bile tract microbiome from recurrent choledocholithiasis patients will likely complement our findings on biliary tract microbiome and clarify some of the implications that arose from our conclusions. Ultimately, these findings have numerous medical implications for both prevention and therapeutics for dCCA or common bile duct stones, warranting further follow-up studies that are needed to verify these findings and move forward.

## 5. Conclusions

We applied 16S sequencing methods to samples collected from the bile of Chinese dCCA patients. Microbial communities of these individuals were significantly different from patients with the new onset of common bile duct stones. We also paid attention to the biliary flora factors associated with stone recurrence. On the other hand, many patient-specific bacteria were found, implying the strong individuality of the biliary microbial community. Taken together, our results provide novel insights into the biliary microbiota with respect to microbial composition, which could be valuable for clinical applications, such as the diagnosis and treatment of bile duct diseases.

## Figures and Tables

**Table 1:** Characteristics of patients.

Characteristic	Group T *n* = 8	Group C *n* = 44	Group P *n* = 16	*p value*
*Gender*				
Male (*n*, %)	3 (37.5%)	18 (40.9%)	9 (56.2%)	0.527
Female (*n*, %)	5 (62.5%)	26 (59.1%)	7 (43.8%)		—
*Age (range)*	72.13 (60–95)	66.98 (44–90)	73.94 (49–92)	0.132
*Diabetes ( n , %)*	0 (0.0%)	10 (22.7%)	4 (25%)	0.303
*Dyslipidemia ( n , %)*	1 (12.5%)	15 (34.1%)	4 (25%)	0.424
*Hypertension (n, %)*	5 (62.5%)	17 (38.6%)	6 (37.5%)	0.426
*Elevated ALT or AST (IU/L) ( n*,* %)*	7 (87.5%)	25 (56.8%)	7 (43.75%)	0.123
*Elevated Tbil and/or Dbil (µmol/L) ( n , %)*	6 (75.0%)	19 (43.2%)	7 (43.75%)	0.241
*Elevated Scr (µmoi/l) ( n , %)*	2 (25.0%)	6 (13.6%)	2 (12.5%)	0.678
*Elevated WBC or NE (10^9/L) ( n , %)*	0 (0.0%)	7 (15.9%)	0 (0.0%)	0.119
*Cholecystolithiasis* (*n, %)*	1 (12.5%)	23 (52.3%)	7 (43.7%)	0.114

AST, Aspartate aminotransferase; ALT, Alanine aminotransferase; Tbil, total bilirubin; Dbil, Direct Bilirubin; Cre, creatinine; WBC, White blood cell; NE, neutrophilicgranulocyte. The dCCA group (Tumor group, T), the new onset of CBD stones group (CBD stones group, C), and recurrent CBD stones group (Post-ERCP CBD stones group, P) denoted as “Group T”, “Group C”, and “Group P”, respectively, in the table.

**Table 2:** Alpha diversity analysis of biliary microbiota in patients with dCCA and the onset of common bile duct stones.

Sample	mean (group_T)	sd (group_T)	mean (group_C)	sd (group_C)	mean (group_P)	sd (group_P)	*p value*
*Observed_OTU*	209.6250	74.5576	165.0682	90.5750	134.2500	66.9423	0.0976
*Chao*	243.4245	73.2746	202.2659	94.6487	181.1733	71.3074	0.2413
*Shannon*	2.8807	1.1989	1.9514	1.1148	1.8863	0.8409	0.0989
*Simpson*	0.7738	0.2312	0.6270	0.2620	0.6687	0.2271	0.2010
*Goodscoverage*	0.9986	0.0006	0.9983	0.0008	0.9983	0.0008	0.8360
*Shannoneven*	0.5355	0.2064	0.3794	0.1877	0.3874	0.1515	0.0887

OTU, operational taxonomic unit; sd, Standard Deviation. The dCCA group (Tumor group, T), the new onset of CBD stones group (CBD stones group, C), and recurrent CBD stones group (Post-ERCP CBD stones group, P) denoted as “Group T”, “Group C”, and “Group P”, respectively, in the table.

**Table 3:** Relative abundance at the phylum level in patients with dCCA and the onset of common bile duct stones.

Phylum	mean (group_T)	sd (group_T)	mean (group_C)	sd (group_C)	*p value*
*Chloroflexi*	0.001255	0.001756	0.000036	0.000142	0.000650
*Gemmatimonadetes*	0.000295	0.000547	0	0	0.000930
*Nitrospirae*	0.000704	0.001973	0	0	0.000930
*Unclassified_Bacteria*	0.118056	0.095475	0.029619	0.074029	0.002429
*Latescibacteria*	0.000600	0.001697	0	0	0.021911
*Planctomycetes*	0.000545	0.001069	0.000002	0.000009	0.039550

The dCCA group (Tumor group, T), the new onset of CBD stones group (CBD stones group, C), and recurrent CBD stones group (Post-ERCP CBD stones group, P) denoted as “Group T”, “Group C”, and “Group P”, respectively, in the table.

**Table 4:** Relative abundance at the genus level in patients with dCCA and the onset of common bile duct stones.

Genus	mean (group_T)	sd (group_T)	mean (group_C)	sd (group_C)	*p* value
*Staphylococcus*	0.1065	0.2852	0.0320	0.1476	0.0002
*Okibacterium*	0.0055	0.0051	0.0011	0.0048	0.0004
*Corynebacterium*	0.0043	0.0071	0.0002	0.0004	0.0009
*Sphingomonas*	0.0047	0.0035	0.0006	0.0014	0.0018
*Stenotrophomonas*	0.0119	0.0110	0.0016	0.0042	0.0021
*Acidovorax*	0.0008	0.0009	0.0002	0.0006	0.0026
*Gemmiger*	0.0049	0.0071	0.0004	0.0010	0.0027
*Providencia*	0.0030	0.0067	0.0009	0.0041	0.0027
*Comamonas*	0.0071	0.0079	0.0006	0.0022	0.0028
*Methylobacterium*	0.0011	0.0009	0.0003	0.0005	0.0029

The dCCA group (Tumor group, T), the new onset of CBD stones group (CBD stones group, C), and recurrent CBD stones group (Post-ERCP CBD stones group, P) denoted as “Group T”, “Group C”, and “Group P”, respectively, in the table.

## Data Availability

The data used to support the findings of this study are available from the corresponding author upon request.

## Supplementary Materials

Supplementary 1 Figure S1: heatmap of bile microbial from all samples. We tested bile in patients with biliary tract tumors and compared all bile microbial organisms in patients with dCCA, the new onset of common bile duct stones and the recurrent choledocholithiasis. A total of 252 bacterial species were identied ,clustering was not consistent with patient grouping.

Supplementary 2 Figure S2: The highest and lowest abundance of Proteobacteria phylum in dCCA patients. The biliary microbiota showed significant inter-individual variation in our test results. For example, the highest abundance of Proteobacteria phylum in one dCCA patient was 74.27% and the lowest was 12.83% in another.

Supplementary 3 Table S1: Alpha diversity analysisof biliary microbiota in patients with dCCA and the onset of common bile duct stones.

Supplementary 4 Table S2: Relative abundance at the genus level in patients with dCCA and the onset of common bile duct stones.

Supplementary 5 Table S3: Relative abundance of 252 bacterial species in patients with dCCA, the new onset of common bile duct stones and the recurrent choledocholithiasis.

Supplementary 6 Table S4: Relative abundance of 252 bacterial species in patients with dCCA, the new onset of common bile duct stones and the recurrent choledocholithiasis.
