# Supplementary material for: A Preliminary Study of Biliary Microbiota in Patients with Bile Duct Stones or Distal Cholangiocarcinoma
Source: Biomed Res Int. 2019 Sep 25;2019:1092563. doi: 10.1155/2019/1092563 (PMC6778921; doi:10.1155/2019/1092563)
Supplement: Supplementary 1 — Figure S1: heatmap of bile microbial from all samples. We tested bile in patients with biliary tract tumors and compared all bile microbial organisms in patients with dCCA, the new onset of common bile duct stones and the recurrent choledocholithiasis. A total of 252 bacterial species were identied ,clustering was not consistent with patient grouping. [file 1092563.f1.docx]

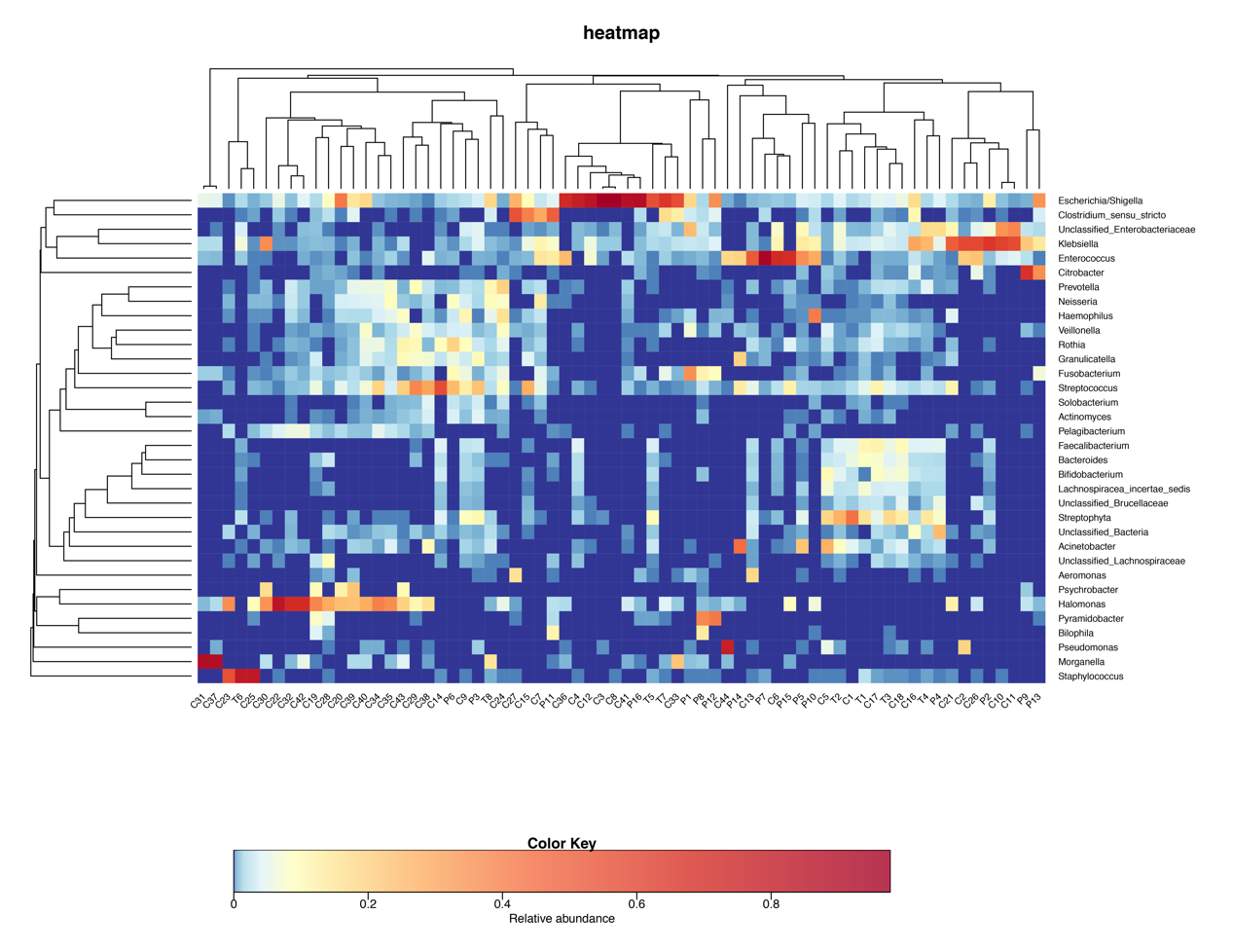


Figure S1: Heatmap of bile microbial from all samples. We tested bile in patientswith biliary tract tumors and compared all bile microbial organisms in patients with dCCA, the new onset of common bile duct stones and the recurrent choledocholithiasis . A total of 252 bacterial species were identied ,clustering was not consistent with patient grouping.
