# Supplementary material for: A Preliminary Study of Biliary Microbiota in Patients with Bile Duct Stones or Distal Cholangiocarcinoma
Source: Biomed Res Int. 2019 Sep 25;2019:1092563. doi: 10.1155/2019/1092563 (PMC6778921; doi:10.1155/2019/1092563)
Supplement: Supplementary 2 — Figure S2: The highest and lowest abundance of Proteobacteria phylum in dCCA patients. The biliary microbiota showed significant inter-individual variation in our test results. For example, the highest abundance of Proteobacteria phylum in one dCCA patient was 74.27% and the lowest was 12.83% in another. [file 1092563.f2.docx]

(a)

Figure S2 The highest and lowest abundance of Proteobacteria phylum in dCCA patients. The biliary microbiota showed significant inter-individual variation in our test results. For example, the highest abundance of Proteobacteria phylum in one dCCA patient was 74.27% and the lowest was 12.83% in another.

(b)
