# Supplementary material for: A Preliminary Study of Biliary Microbiota in Patients with Bile Duct Stones or Distal Cholangiocarcinoma
Source: Biomed Res Int. 2019 Sep 25;2019:1092563. doi: 10.1155/2019/1092563 (PMC6778921; doi:10.1155/2019/1092563)
Supplement: Supplementary 3 — Table S1: Alpha diversity analysisof biliary microbiota in patients with dCCA and the onset of common bile duct stones. [file 1092563.f3.docx]

Table S1 Alpha diversity analysisof biliary microbiota in patients with dCCA and the onset of common bile duct stones

|  | **mean(group_T)** | **sd(group_T)** | **mean(group_C)** | **sd(group_C)** | *p* value |
| --- | --- | --- | --- | --- | --- |
| **Observed_OTU** | 209.625 | 74.557 | 165.068 | 90.575 | 0.159 |
| **Chao** | 243.424 | 73.274 | 202.265 | 94.648 | 0.243 |
| **Shannon** | 2.880 | 1.1989 | 1.951 | 1.114 | 0.036 |
| **Simpson** | 0.773 | 0.231 | 0.627 | 0.262 | 0.077 |
| **GoodsCoverage** | 0.998 | 0.001 | 0.998 | 0.001 | 0.657 |
| **shannoneven** | 0.535 | 0.206 | 0.379 | 0.187 | 0.030 |
| OTU,operational taxonomic unit;sd, Standard Deviation.The dCCA group(Tumor group, T) and the new onset of CBD stones group (CBD stones group, C) denoted as “Group T”and “Group C” ,respectively, in the table. | | | | | |
