# Supplementary material for: A Preliminary Study of Biliary Microbiota in Patients with Bile Duct Stones or Distal Cholangiocarcinoma
Source: Biomed Res Int. 2019 Sep 25;2019:1092563. doi: 10.1155/2019/1092563 (PMC6778921; doi:10.1155/2019/1092563)
Supplement: Supplementary 4 — Table S2: Relative abundance at the genus level in patients with dCCA and the onset of common bile duct stones. [file 1092563.f4.docx]

Table S2：Relative abundance at the genus level in patients with dCCA and the onset of common bile duct stones

| **genus** | **mean(group_C)** | **sd(group_C)** | **mean(group_T)** | **sd(group_T)** |
| --- | --- | --- | --- | --- |
| **Escherichia/Shigella** | 0.17324200 | 0.29991220 | 0.19063930 | 0.26114920 |
| **Unclassified_Bacteria** | 0.02961934 | 0.07402864 | 0.11805590 | 0.09547475 |
| **Staphylococcus** | 0.03198084 | 0.14760300 | 0.10648050 | 0.28520160 |
| **Klebsiella** | 0.09433479 | 0.18602600 | 0.06149356 | 0.08144658 |
| **Unclassified_Enterobacteriaceae** | 0.02589709 | 0.07697471 | 0.04704342 | 0.05379589 |
| **Faecalibacterium** | 0.01031778 | 0.02452319 | 0.03652084 | 0.03637420 |
| **Clostridium_sensu_stricto** | 0.03616825 | 0.10931580 | 0.02640192 | 0.05847170 |
| **Acinetobacter** | 0.01312261 | 0.04327418 | 0.02531639 | 0.02645988 |
| **Bacteroides** | 0.00868129 | 0.01774361 | 0.02444905 | 0.02884426 |
| **Prevotella** | 0.01783072 | 0.03460037 | 0.02287803 | 0.03693528 |
| **Streptococcus** | 0.06477298 | 0.11766820 | 0.02019420 | 0.01898029 |
| **Lachnospiracea_incertae_sedis** | 0.00519211 | 0.01278461 | 0.01971962 | 0.02428231 |
| **Bifidobacterium** | 0.00906215 | 0.02222506 | 0.01969234 | 0.02405064 |
| **Morganella** | 0.04993851 | 0.18827380 | 0.01968143 | 0.05315323 |
| **Neisseria** | 0.01403309 | 0.02735427 | 0.01336461 | 0.03252814 |
| **Fusobacterium** | 0.00809811 | 0.01398138 | 0.01295003 | 0.01774901 |
| **Stenotrophomonas** | 0.00160276 | 0.00423207 | 0.01192450 | 0.01101241 |
| **Unclassified_Brucellaceae** | 0.00375498 | 0.00952952 | 0.00924613 | 0.00968071 |
| **Haemophilus** | 0.01507647 | 0.03118028 | 0.00838970 | 0.01728605 |
| **Rothia** | 0.01688850 | 0.02989044 | 0.00747327 | 0.00980909 |
| **Veillonella** | 0.01279234 | 0.02149497 | 0.00734781 | 0.00524567 |
| **Enterococcus** | 0.05886379 | 0.14074330 | 0.00710234 | 0.00498773 |
| **Comamonas** | 0.00060302 | 0.00223703 | 0.00709143 | 0.00785238 |
| **Anoxybacillus** | 0.00149465 | 0.00366281 | 0.00697687 | 0.00638981 |
| **Clostridium_XlVa** | 0.00255589 | 0.00540779 | 0.00679140 | 0.00665323 |
| **Halomonas** | 0.13638640 | 0.21235520 | 0.00585861 | 0.01418771 |
| **Geobacillus** | 0.00085196 | 0.00234907 | 0.00578224 | 0.00768031 |
| **Okibacterium** | 0.00110884 | 0.00483714 | 0.00549313 | 0.00514896 |
| **Gemmiger** | 0.00035011 | 0.00099427 | 0.00494218 | 0.00711735 |
| **Porphyromonas** | 0.00234662 | 0.00504228 | 0.00477853 | 0.01043265 |
| **Sphingomonas** | 0.00062881 | 0.00140309 | 0.00470216 | 0.00352249 |
| **Unclassified_Ruminococcaceae** | 0.00248944 | 0.00710742 | 0.00456033 | 0.00595936 |
| **Alloprevotella** | 0.00247952 | 0.00491901 | 0.00446214 | 0.01132916 |
| **Corynebacterium** | 0.00017158 | 0.00037462 | 0.00430395 | 0.00711628 |
| **Clostridium_XVIII** | 0.00088172 | 0.00215760 | 0.00402029 | 0.00417557 |
| **Ruminococcus** | 0.00248448 | 0.00696341 | 0.00378573 | 0.00377517 |
| **Granulicatella** | 0.01257116 | 0.02260737 | 0.00350753 | 0.00487715 |
| **Ruminococcus2** | 0.00085196 | 0.00199295 | 0.00333297 | 0.00477665 |
| **Brevundimonas** | 0.00077262 | 0.00252997 | 0.00327297 | 0.00357098 |
| **Roseburia** | 0.00070914 | 0.00184169 | 0.00325660 | 0.00525681 |
| **Akkermansia** | 0.00138655 | 0.00319212 | 0.00321296 | 0.00336885 |
| **Parabacteroides** | 0.00111975 | 0.00270021 | 0.00316387 | 0.00468882 |
| **Aquabacterium** | 0.00032432 | 0.00062588 | 0.00301113 | 0.00329397 |
| **Providencia** | 0.00087180 | 0.00405650 | 0.00299476 | 0.00667177 |
| **Unclassified_Clostridiales** | 0.00063872 | 0.00128608 | 0.00295658 | 0.00423620 |
| **Barnesiella** | 0.00208379 | 0.00696932 | 0.00292385 | 0.00270877 |
| **Unclassified_Lachnospiraceae** | 0.00448793 | 0.01432424 | 0.00276566 | 0.00328238 |
| **Thermaerobacter** | 0.00020332 | 0.00054562 | 0.00232926 | 0.00319766 |
| **Actinomyces** | 0.00268978 | 0.00591270 | 0.00224744 | 0.00256048 |
| **Dialister** | 0.00042747 | 0.00086379 | 0.00219834 | 0.00397493 |
| **Leptotrichia** | 0.00157102 | 0.00270753 | 0.00213834 | 0.00339276 |
| **Megamonas** | 0.00098883 | 0.00336512 | 0.00193105 | 0.00391644 |
| **Blautia** | 0.00050285 | 0.00105818 | 0.00183832 | 0.00202193 |
| **Gemella** | 0.00197965 | 0.00494147 | 0.00179468 | 0.00229043 |
| **Atopobium** | 0.00239323 | 0.00561412 | 0.00160921 | 0.00247154 |
| **Oscillibacter** | 0.00073295 | 0.00206899 | 0.00146193 | 0.00266992 |
| **Collinsella** | 0.00030052 | 0.00075874 | 0.00145647 | 0.00261473 |
| **Oribacterium** | 0.00147779 | 0.00342832 | 0.00141283 | 0.00204580 |
| **Saccharibacteria_genera_incertae_sedis** | 0.00092536 | 0.00182619 | 0.00135283 | 0.00290129 |
| **Solobacterium** | 0.00292187 | 0.00776566 | 0.00135283 | 0.00220653 |
| **Lachnoanaerobaculum** | 0.00084006 | 0.00184611 | 0.00133646 | 0.00308092 |
| **Citrobacter** | 0.00363101 | 0.01112844 | 0.00129282 | 0.00124111 |
| **Peptostreptococcus** | 0.00132307 | 0.00379382 | 0.00128191 | 0.00243030 |
| **Pseudomonas** | 0.02398290 | 0.11949580 | 0.00118372 | 0.00118562 |
| **Methylobacterium** | 0.00025093 | 0.00050033 | 0.00111281 | 0.00086810 |
| **Clostridium_XlVb** | 0.00023010 | 0.00058973 | 0.00105280 | 0.00148272 |
| **Alistipes** | 0.00050384 | 0.00109066 | 0.00105280 | 0.00144085 |
| **Longilinea** | 0.00000793 | 0.00003017 | 0.00099825 | 0.00131860 |
| **Lactobacillus** | 0.00067245 | 0.00165451 | 0.00096552 | 0.00126295 |
| **Clostridium_XI** | 0.00056434 | 0.00158154 | 0.00092734 | 0.00084096 |
| **Aggregatibacter** | 0.00112074 | 0.00389084 | 0.00089461 | 0.00246024 |
| **Campylobacter** | 0.00151449 | 0.00312763 | 0.00085097 | 0.00162483 |
| **Unclassified_Porphyromonadaceae** | 0.00136770 | 0.00571944 | 0.00081824 | 0.00089609 |
| **Megasphaera** | 0.00103743 | 0.00227007 | 0.00080188 | 0.00112691 |
| **Unclassified_Actinomycetales** | 0.00022018 | 0.00052922 | 0.00076915 | 0.00097635 |
| **Selenomonas** | 0.00030349 | 0.00059694 | 0.00075824 | 0.00153102 |
| **Unclassified_Alphaproteobacteria** | 0.00002182 | 0.00014474 | 0.00075278 | 0.00212919 |
| **Acidovorax** | 0.00017952 | 0.00059748 | 0.00075278 | 0.00085029 |
| **Anaerostipes** | 0.00023407 | 0.00066623 | 0.00074733 | 0.00119795 |
| **Dorea** | 0.00015075 | 0.00036298 | 0.00072551 | 0.00138785 |
| **SR1_genera_incertae_sedis** | 0.00033821 | 0.00117844 | 0.00071460 | 0.00132938 |
| **Nitrospira** | 0.00000000 | 0.00000000 | 0.00070369 | 0.00197276 |
| **Aeromonas** | 0.00811001 | 0.03478122 | 0.00069823 | 0.00064475 |
| **Butyricicoccus** | 0.00037788 | 0.00083646 | 0.00065459 | 0.00084040 |
| **Clostridium_IV** | 0.00040763 | 0.00141646 | 0.00064914 | 0.00072215 |
| **Treponema** | 0.00015175 | 0.00040431 | 0.00063277 | 0.00178975 |
| **Phascolarctobacterium** | 0.00021522 | 0.00051017 | 0.00063277 | 0.00079309 |
| **Rhizobium** | 0.00005951 | 0.00013962 | 0.00061641 | 0.00058336 |
| **Capnocytophaga** | 0.00156011 | 0.00501237 | 0.00061641 | 0.00163890 |
| **Unclassified_Comamonadaceae** | 0.00001190 | 0.00006679 | 0.00061095 | 0.00169305 |
| **Pyramidobacter** | 0.00308948 | 0.01389763 | 0.00061095 | 0.00133021 |
| **Eubacterium** | 0.00127844 | 0.00276822 | 0.00060550 | 0.00098066 |
| **Latescibacteria_genera_incertae_sedis** | 0.00000000 | 0.00000000 | 0.00060004 | 0.00169718 |
| **Spartobacteria_genera_incertae_sedis** | 0.00000397 | 0.00002632 | 0.00060004 | 0.00108555 |
| **Micrococcus** | 0.00002380 | 0.00006132 | 0.00060004 | 0.00159265 |
| **Unclassified_Bradyrhizobiaceae** | 0.00006943 | 0.00016228 | 0.00058913 | 0.00072349 |
| **Derxia** | 0.00036102 | 0.00112756 | 0.00058368 | 0.00144584 |
| **Serratia** | 0.00055244 | 0.00297479 | 0.00054549 | 0.00131520 |
| **Unclassified_Phyllobacteriaceae** | 0.00389086 | 0.00684130 | 0.00054549 | 0.00050029 |
| **Caulobacter** | 0.00006943 | 0.00019041 | 0.00054004 | 0.00064007 |
| **Nesterenkonia** | 0.00136572 | 0.00216059 | 0.00054004 | 0.00132138 |
| **Anaerococcus** | 0.00002579 | 0.00007309 | 0.00052913 | 0.00078184 |
| **Unclassified_Pasteurellaceae** | 0.00050384 | 0.00200919 | 0.00050185 | 0.00074022 |
| **Acetivibrio** | 0.00126753 | 0.00729551 | 0.00050185 | 0.00083324 |
| **Streptobacillus** | 0.00014580 | 0.00039393 | 0.00049094 | 0.00138860 |
| **Flavonifractor** | 0.00068831 | 0.00198933 | 0.00048549 | 0.00066733 |
| **Finegoldia** | 0.00016266 | 0.00048871 | 0.00048003 | 0.00070018 |
| **Parasutterella** | 0.00046218 | 0.00103657 | 0.00048003 | 0.00070405 |
| **Unclassified_Betaproteobacteria** | 0.00000099 | 0.00000658 | 0.00043640 | 0.00123431 |
| **Rhodococcus** | 0.00008232 | 0.00031794 | 0.00043640 | 0.00060828 |
| **Massilia** | 0.00016662 | 0.00046625 | 0.00043640 | 0.00059653 |
| **Sediminibacterium** | 0.00017456 | 0.00076245 | 0.00042549 | 0.00050557 |
| **Filifactor** | 0.00055839 | 0.00195102 | 0.00040912 | 0.00088361 |
| **Unclassified_Rhodobacteraceae** | 0.00003273 | 0.00014795 | 0.00040367 | 0.00060277 |
| **Rheinheimera** | 0.00003670 | 0.00021707 | 0.00040367 | 0.00042932 |
| **Hydrogenophilus** | 0.00000793 | 0.00002357 | 0.00039276 | 0.00105846 |
| **Coprobacillus** | 0.00000000 | 0.00000000 | 0.00038185 | 0.00108002 |
| **Peptoniphilus** | 0.00000595 | 0.00002915 | 0.00037639 | 0.00066794 |
| **Bilophila** | 0.00114653 | 0.00618031 | 0.00037094 | 0.00044381 |
| **Candidatus_Kuenenia** | 0.00000198 | 0.00000920 | 0.00035457 | 0.00100288 |
| **Desulfomicrobium** | 0.00000694 | 0.00003987 | 0.00034912 | 0.00095267 |
| **Odoribacter** | 0.00014282 | 0.00039555 | 0.00033821 | 0.00043374 |
| **Unclassified_Actinobacteria** | 0.00000000 | 0.00000000 | 0.00032730 | 0.00092573 |
| **Eggerthella** | 0.00003868 | 0.00010102 | 0.00032730 | 0.00062286 |
| **Pelomonas** | 0.00007538 | 0.00022945 | 0.00032184 | 0.00037896 |
| **Tannerella** | 0.00010216 | 0.00027823 | 0.00031639 | 0.00056743 |
| **Moryella** | 0.00088767 | 0.00220670 | 0.00031639 | 0.00057647 |
| **Gp4** | 0.00015274 | 0.00073792 | 0.00031093 | 0.00084473 |
| **Unclassified_Desulfovibrionaceae** | 0.00051276 | 0.00165531 | 0.00030548 | 0.00065853 |
| **Gemmatimonas** | 0.00000000 | 0.00000000 | 0.00029457 | 0.00054742 |
| **Hafnia** | 0.00051872 | 0.00272654 | 0.00029457 | 0.00033783 |
| **Unclassified_Bacteroidales** | 0.00006943 | 0.00018354 | 0.00028911 | 0.00047344 |
| **Proteus** | 0.00014084 | 0.00040315 | 0.00027820 | 0.00044622 |
| **Achromobacter** | 0.00018944 | 0.00073999 | 0.00027820 | 0.00045766 |
| **Opitutus** | 0.00000000 | 0.00000000 | 0.00025638 | 0.00072516 |
| **Unclassified_Deltaproteobacteria** | 0.00000000 | 0.00000000 | 0.00024547 | 0.00069430 |
| **Pelagibacterium** | 0.00889850 | 0.01552817 | 0.00024547 | 0.00056656 |
| **Alkanindiges** | 0.00000496 | 0.00002149 | 0.00023456 | 0.00064599 |
| **Enhydrobacter** | 0.00002480 | 0.00006547 | 0.00022911 | 0.00039362 |
| **Sphingobium** | 0.00010414 | 0.00025950 | 0.00022911 | 0.00046345 |
| **Niastella** | 0.00000000 | 0.00000000 | 0.00022365 | 0.00063259 |
| **Proteiniphilum** | 0.00000000 | 0.00000000 | 0.00022365 | 0.00063259 |
| **Brevibacterium** | 0.00000099 | 0.00000658 | 0.00022365 | 0.00037932 |
| **Erysipelotrichaceae_incertae_sedis** | 0.00018348 | 0.00047326 | 0.00022365 | 0.00040967 |
| **Desulfovibrio** | 0.00032035 | 0.00201174 | 0.00022365 | 0.00063259 |
| **Gp2** | 0.00000000 | 0.00000000 | 0.00021274 | 0.00060173 |
| **Unclassified_Burkholderiales** | 0.00000198 | 0.00001316 | 0.00019638 | 0.00055544 |
| **Candidatus_Brocadia** | 0.00000000 | 0.00000000 | 0.00019092 | 0.00054001 |
| **Sporobacter** | 0.00010712 | 0.00028801 | 0.00019092 | 0.00024234 |
| **Propionibacterium** | 0.00003868 | 0.00009881 | 0.00018547 | 0.00033540 |
| **Unclassified_Rhodocyclaceae** | 0.00000000 | 0.00000000 | 0.00016365 | 0.00046287 |
| **Unclassified_Chloroflexi** | 0.00001984 | 0.00013158 | 0.00016365 | 0.00039706 |
| **Coprococcus** | 0.00002876 | 0.00009409 | 0.00016365 | 0.00035125 |
| **Pseudoflavonifractor** | 0.00024002 | 0.00077818 | 0.00016365 | 0.00024101 |
| **Pontibacter** | 0.00000000 | 0.00000000 | 0.00015819 | 0.00044744 |
| **Unclassified_Chitinophagaceae** | 0.00000000 | 0.00000000 | 0.00015819 | 0.00044744 |
| **Rhizobacter** | 0.00000496 | 0.00003289 | 0.00015819 | 0.00029315 |
| **Catonella** | 0.00056434 | 0.00111432 | 0.00015274 | 0.00021123 |
| **Parvimonas** | 0.00058814 | 0.00141610 | 0.00015274 | 0.00028664 |
| **Hydrogenophaga** | 0.00000298 | 0.00001974 | 0.00013637 | 0.00031850 |
| **Empedobacter** | 0.00000893 | 0.00003594 | 0.00013637 | 0.00033434 |
| **Bosea** | 0.00003075 | 0.00014779 | 0.00013637 | 0.00021050 |
| **Unclassified_Flavobacteriaceae** | 0.00013687 | 0.00026480 | 0.00013637 | 0.00036841 |
| **Cupriavidus** | 0.00001884 | 0.00007253 | 0.00013092 | 0.00023558 |
| **Roseomonas** | 0.00000198 | 0.00001316 | 0.00012001 | 0.00033944 |
| **Weissella** | 0.00001488 | 0.00004885 | 0.00012001 | 0.00022944 |
| **Rubellimicrobium** | 0.00000397 | 0.00001580 | 0.00011455 | 0.00032401 |
| **Dietzia** | 0.00000793 | 0.00005263 | 0.00010910 | 0.00030858 |
| **Unclassified_Xanthobacteraceae** | 0.00000298 | 0.00001974 | 0.00010364 | 0.00020856 |
| **Unclassified_Anaerolineaceae** | 0.00000793 | 0.00005263 | 0.00009273 | 0.00026229 |
| **Butyrivibrio** | 0.00011108 | 0.00031936 | 0.00008728 | 0.00014936 |
| **Lysobacter** | 0.00001686 | 0.00007998 | 0.00007637 | 0.00011605 |
| **Erythrobacter** | 0.00003967 | 0.00014391 | 0.00007637 | 0.00021600 |
| **Murdochiella** | 0.00000298 | 0.00001974 | 0.00007091 | 0.00014367 |
| **Unclassified_Clostridiales_Incertae_Sedis_XI** | 0.00002579 | 0.00007489 | 0.00007091 | 0.00020058 |
| **Unclassified_Myxococcales** | 0.00000298 | 0.00001457 | 0.00006546 | 0.00008728 |
| **Peptococcus** | 0.00002083 | 0.00007259 | 0.00006546 | 0.00018515 |
| **Ralstonia** | 0.00002182 | 0.00005024 | 0.00006546 | 0.00016821 |
| **Paracoccus** | 0.00008728 | 0.00049283 | 0.00006546 | 0.00009897 |
| **Johnsonella** | 0.00012398 | 0.00068459 | 0.00006546 | 0.00016821 |
| **Helicobacter** | 0.00036102 | 0.00081242 | 0.00006546 | 0.00010168 |
| **Psychrobacter** | 0.01601075 | 0.04806267 | 0.00006546 | 0.00012121 |
| **Unclassified_Firmicutes** | 0.00000198 | 0.00001316 | 0.00006000 | 0.00016972 |
| **Unclassified_Bacteroidetes** | 0.00001587 | 0.00006793 | 0.00006000 | 0.00016972 |
| **Allisonella** | 0.00007736 | 0.00044661 | 0.00005455 | 0.00010626 |
| **Unclassified_Desulfovibrionales** | 0.00000000 | 0.00000000 | 0.00004909 | 0.00013886 |
| **Vampirovibrio** | 0.00001984 | 0.00007134 | 0.00004909 | 0.00009165 |
| **Rhodobacter** | 0.00000000 | 0.00000000 | 0.00004364 | 0.00010689 |
| **Anaerovorax** | 0.00014679 | 0.00035876 | 0.00004364 | 0.00012343 |
| **Chelatococcus** | 0.00000000 | 0.00000000 | 0.00003818 | 0.00009165 |
| **Arthrobacter** | 0.00000099 | 0.00000658 | 0.00003818 | 0.00010800 |
| **Holdemania** | 0.00007339 | 0.00030711 | 0.00003818 | 0.00004914 |
| **Abiotrophia** | 0.00054153 | 0.00158906 | 0.00003818 | 0.00007536 |
| **Schwartzia** | 0.00001289 | 0.00007325 | 0.00003273 | 0.00009257 |
| **Paludibacter** | 0.00002777 | 0.00006922 | 0.00003273 | 0.00009257 |
| **Kingella** | 0.00000893 | 0.00002591 | 0.00002182 | 0.00006172 |
| **Cloacibacterium** | 0.00001488 | 0.00005235 | 0.00002182 | 0.00006172 |
| **Unclassified_Sutterellaceae** | 0.00001686 | 0.00010532 | 0.00002182 | 0.00006172 |
| **Novosphingobium** | 0.00001785 | 0.00005008 | 0.00002182 | 0.00004040 |
| **Eikenella** | 0.00005157 | 0.00013319 | 0.00002182 | 0.00006172 |
| **Mobiluncus** | 0.00006645 | 0.00035208 | 0.00002182 | 0.00003299 |
| **Shewanella** | 0.00016960 | 0.00078688 | 0.00002182 | 0.00003299 |
| **Unclassified_Veillonellaceae** | 0.00001091 | 0.00003895 | 0.00001091 | 0.00003086 |
| **Marvinbryantia** | 0.00002876 | 0.00008467 | 0.00001091 | 0.00002020 |
| **Anaerotruncus** | 0.00003571 | 0.00009928 | 0.00001091 | 0.00003086 |
| **Vibrio** | 0.00004364 | 0.00013210 | 0.00001091 | 0.00002020 |
| **Terrimonas** | 0.00000000 | 0.00000000 | 0.00000545 | 0.00001543 |
| **Dyadobacter** | 0.00000099 | 0.00000658 | 0.00000545 | 0.00001543 |
| **Unclassified_Proteobacteria** | 0.00001884 | 0.00012500 | 0.00000545 | 0.00001543 |
| **Butyricimonas** | 0.00004066 | 0.00013190 | 0.00000545 | 0.00001543 |
| **Mogibacterium** | 0.00008034 | 0.00031171 | 0.00000545 | 0.00001543 |
| **Unclassified_Prevotellaceae** | 0.00013290 | 0.00062450 | 0.00000545 | 0.00001543 |
| **Porphyrobacter** | 0.00000000 | 0.00000000 | 0.00000000 | 0.00000000 |
| **Aminicenantes_genera_incertae_sedis** | 0.00000099 | 0.00000658 | 0.00000000 | 0.00000000 |
| **Unclassified_Rhizobiales** | 0.00000298 | 0.00001457 | 0.00000000 | 0.00000000 |
| **Deinococcus** | 0.00000397 | 0.00002632 | 0.00000000 | 0.00000000 |
| **Pseudoxanthomonas** | 0.00000496 | 0.00002698 | 0.00000000 | 0.00000000 |
| **Propionicicella** | 0.00000595 | 0.00003947 | 0.00000000 | 0.00000000 |
| **Unclassified_Clostridiaceae_1** | 0.00000694 | 0.00003514 | 0.00000000 | 0.00000000 |
| **Desulfobulbus** | 0.00000793 | 0.00003556 | 0.00000000 | 0.00000000 |
| **Hallella** | 0.00000793 | 0.00005263 | 0.00000000 | 0.00000000 |
| **Wolinella** | 0.00000793 | 0.00003556 | 0.00000000 | 0.00000000 |
| **Anaeroplasma** | 0.00000893 | 0.00004057 | 0.00000000 | 0.00000000 |
| **Pedobacter** | 0.00000992 | 0.00005942 | 0.00000000 | 0.00000000 |
| **Craurococcus** | 0.00001190 | 0.00004645 | 0.00000000 | 0.00000000 |
| **Subdoligranulum** | 0.00001389 | 0.00006155 | 0.00000000 | 0.00000000 |
| **Gp1** | 0.00001488 | 0.00009868 | 0.00000000 | 0.00000000 |
| **Mycoplasma** | 0.00002083 | 0.00007074 | 0.00000000 | 0.00000000 |
| **Azospira** | 0.00002182 | 0.00009245 | 0.00000000 | 0.00000000 |
| **Anaeroglobus** | 0.00002281 | 0.00005023 | 0.00000000 | 0.00000000 |
| **Lactococcus** | 0.00002678 | 0.00013998 | 0.00000000 | 0.00000000 |
| **Edwardsiella** | 0.00003372 | 0.00013036 | 0.00000000 | 0.00000000 |
| **Paraprevotella** | 0.00003868 | 0.00014218 | 0.00000000 | 0.00000000 |
| **Unclassified_Lactobacillales** | 0.00003967 | 0.00016672 | 0.00000000 | 0.00000000 |
| **Olsenella** | 0.00004066 | 0.00014501 | 0.00000000 | 0.00000000 |
| **Unclassified_Peptococcaceae_1** | 0.00004066 | 0.00019389 | 0.00000000 | 0.00000000 |
| **Caldisericum** | 0.00004166 | 0.00027631 | 0.00000000 | 0.00000000 |
| **Cardiobacterium** | 0.00004265 | 0.00009149 | 0.00000000 | 0.00000000 |
| **Unclassified_Leptotrichiaceae** | 0.00004364 | 0.00012162 | 0.00000000 | 0.00000000 |
| **Roseococcus** | 0.00004463 | 0.00029605 | 0.00000000 | 0.00000000 |
| **Peptostreptococcaceae_incertae_sedis** | 0.00005157 | 0.00030905 | 0.00000000 | 0.00000000 |
| **Paraeggerthella** | 0.00006348 | 0.00037486 | 0.00000000 | 0.00000000 |
| **Unclassified_Corynebacteriaceae** | 0.00006744 | 0.00016177 | 0.00000000 | 0.00000000 |
| **Bulleidia** | 0.00008034 | 0.00048017 | 0.00000000 | 0.00000000 |
| **Unclassified_Synergistaceae** | 0.00011902 | 0.00056023 | 0.00000000 | 0.00000000 |
| **Turicibacter** | 0.00012398 | 0.00048822 | 0.00000000 | 0.00000000 |
| **Actinotalea** | 0.00013885 | 0.00091434 | 0.00000000 | 0.00000000 |
| **Alloscardovia** | 0.00015472 | 0.00090696 | 0.00000000 | 0.00000000 |
| **Allobaculum** | 0.00017059 | 0.00074201 | 0.00000000 | 0.00000000 |
| **Blastococcus** | 0.00020530 | 0.00112225 | 0.00000000 | 0.00000000 |
| **Phocaeicola** | 0.00021522 | 0.00135380 | 0.00000000 | 0.00000000 |
| **Nocardioides** | 0.00075278 | 0.00412720 | 0.00000000 | 0.00000000 |
| The dCCA group(Tumor group, T) and the new onset of CBD stones group (CBD stones group, C) denoted as “Group T” and “Group C” ,respectively, in the table. | | | | |
