# Supplementary material for: A Preliminary Study of Biliary Microbiota in Patients with Bile Duct Stones or Distal Cholangiocarcinoma
Source: Biomed Res Int. 2019 Sep 25;2019:1092563. doi: 10.1155/2019/1092563 (PMC6778921; doi:10.1155/2019/1092563)
Supplement: Supplementary 6 — Table S4: Relative abundance of 252 bacterial species in patients with dCCA, the new onset of common bile duct stones and the recurrent choledocholithiasis. [file 1092563.f6.docx]

TABLE S4: Alpha diversity analysisof biliary microbiota in patients with the onset of common bile duct stones and the recurrent choledocholithiasis.

| **Sample** | **mean(group_C)** | **sd(group_C)** | **mean(group_P)** | **sd(group_P)** | *p* value |
| --- | --- | --- | --- | --- | --- |
| **Observed_OTU** | 165.0682 | 90.5750 | 134.2500 | 66.9423 | 0.1652 |
| **Chao** | 202.2659 | 94.6487 | 181.1733 | 71.3074 | 0.4408 |
| **Shannon** | 1.9514 | 1.1148 | 1.8863 | 0.8409 | 0.9934 |
| **Simpson** | 0.6270 | 0.2620 | 0.6687 | 0.2271 | 0.7096 |
| **GoodsCoverage** | 0.9983 | 0.0008 | 0.9983 | 0.0008 | 0.7067 |
| **shannoneven** | 0.3794 | 0.1877 | 0.3874 | 0.1515 | 0.8104 |
| OTU,operational taxonomic unit;sd, Standard Deviation. The new onset of CBD stones group (CBD stones group, C) and recurrent CBD stones group(Post-ERCP CBD stones group, P) denoted as “Group C” and “Group P” ,respectively, in the table. | | | | | |
